# Association between administered oxygen, arterial partial oxygen pressure and mortality in mechanically ventilated intensive care unit patients

**DOI:** 10.1186/cc7150

**Published:** 2008-12-10

**Authors:** Evert de Jonge, Linda Peelen, Peter J Keijzers, Hans Joore, Dylan de Lange, Peter HJ van der Voort, Robert J Bosman, Ruud AL de Waal, Ronald Wesselink, Nicolette F de Keizer

**Affiliations:** 1 Department of Intensive Care, Academic Medical Center, Meibergdreef 9, 1105 AZ Amsterdam, The Netherlands; 2 Department of Medical Informatics, Academic Medical Center, Meibergdreef 9, 1105 AZ Amsterdam, The Netherlands; 3 Department of Epidemiology, Julius Center for Health Sciences and Primary Care, University Medical Center, Heidelberglaan 100, 3584 CX Utrecht, The Netherlands; 4 Intensive Care, University Medical Center, Heidelberglaan 100, 3584 CX, Utrecht, The Netherlands; 5 Intensive Care, Onze Lieve Vrouwe Gasthuis, Oosterpark 91091 AC Amsterdam, The Netherlands; 6 Intensive Care, Amphia Medical Center, Molengracht 21, 4818 CK Breda, The Netherlands; 7 Intensive Care, St. Antonius Hospital Koekoekslaan 1, 3435 CM Nieuwegein, The Netherlands

## Abstract

**Introduction:**

The aim of this study was to investigate whether in-hospital mortality was associated with the administered fraction of oxygen in inspired air (FiO_2_) and achieved arterial partial pressure of oxygen (PaO_2_).

**Methods:**

This was a retrospective, observational study on data from the first 24 h after admission from 36,307 consecutive patients admitted to 50 Dutch intensive care units (ICUs) and treated with mechanical ventilation. Oxygenation data from all admission days were analysed in a subset of 3,322 patients in 5 ICUs.

**Results:**

Mean PaO_2 _and FiO_2 _in the first 24 h after ICU admission were 13.2 kPa (standard deviation (SD) 6.5) and 50% (SD 20%) respectively. Mean PaO_2 _and FiO_2 _from all admission days were 12.4 kPa (SD 5.5) and 53% (SD 18). Focusing on oxygenation in the first 24 h of admission, in-hospital mortality was shown to be linearly related to FiO_2 _value and had a U-shaped relationship with PaO_2 _(both lower and higher PaO_2 _values were associated with a higher mortality), independent of each other and of Simplified Acute Physiology Score (SAPS) II, age, admission type, reduced Glasgow Coma Scale (GCS) score, and individual ICU. Focusing on the entire ICU stay, in-hospital mortality was independently associated with mean FiO_2 _during ICU stay and with the lower two quintiles of mean PaO_2 _value during ICU stay.

**Conclusions:**

Actually achieved PaO_2 _values in ICU patients in The Netherlands are higher than generally recommended in the literature. High FiO_2_, and both low PaO_2 _and high PaO_2 _in the first 24 h after admission are independently associated with in-hospital mortality in ICU patients. Future research should study whether this association is causal or merely a reflection of differences in severity of illness insufficiently corrected for in the multivariate analysis.

## Introduction

It is generally acknowledged that mechanical ventilation may cause or exacerbate lung damage in critically ill patients with acute lung injury (ALI) or acute respiratory distress syndrome (ARDS). Many studies have examined the effects of different settings of ventilation, such as low vs high tidal volumes, prone positioning and high-frequency oscillation on outcome of intensive care unit (ICU) patients [1]. Lung-protective mechanical ventilation strategies in patients with ALI/ARDS, applying lower tidal volumes and sufficient levels of positive end expiratory pressure (PEEP) [2,3], have been shown to improve outcome.

The mode of mechanical ventilation and the oxygenation targets may influence the outcome for patients. Traditionally, arterial oxygen concentration (measured as partial oxygen pressure, PaO_2_) and oxygen saturation by pulse oximetry are used as targets. Common recommendations for oxygenation propose PaO_2 _values to be between 7.3 and 10.6 kPa [2,4]. The deleterious effects of hypoxia are well known and physicians may be mostly concerned about avoiding hypoxia and give additional oxygen 'to be on the safe side'. Hyperoxia, however, is also to be avoided as oxygen may be toxic. First, it is long known that high fraction of oxygen in inspired air (FiO_2_) may be toxic for the lungs. In animals, prolonged hyperoxia causes histopathological changes similar to those seen in ARDS [5]. Baboons exposed to 100% oxygen demonstrated a progressive reduction in forced vital capacity and functional residual capacity [6] and proliferative epithelial changes and interstitial fibrosis [7]. In healthy humans, exposure to 100% oxygen may lead to atelectasis, impaired mucocilliary clearance and tracheobronchitis, alveolar protein leakage and enhanced expression of leukotrienes by alveolar macrophages and increases in alveolar neutrophils [8]. Apart from its effects on the lungs, oxygen may also lead to systemic toxicity. It has been associated with an increase in vascular resistance and a decrease in cardiac output [9]. Hyperoxia may result in the generation of central nervous system, hepatic and pulmonary free radicals. Cardiopulmonary resuscitation following cardiac arrest in a canine model is associated with a worsened neurologic outcome when performed in the presence of hyperoxia vs normoxia [8,10].

The aim of the present study was to describe the present oxygenation targets applied in ICUs in The Netherlands, and to determine whether outcome of ICU patients was associated with differences in administered oxygen (FiO_2_) or achieved arterial PaO_2_.

## Materials and methods

### Patient data

This study is based on retrospective analysis of all consecutive patients admitted between 1 January 1999 and 30 June 2006 to the ICUs of 50 university, teaching and non-teaching hospitals in The Netherlands who were on mechanical ventilation within the first 24 h after ICU admission. Data were collected as part of the Dutch National Intensive Care Evaluation (NICE) registry. Within this registry data collection takes place in a standardised manner according to strict definitions and is subject to stringent data quality checks, which has been shown to result in a high quality of data [11]. The data have been encrypted in a way that all patient identifying information, such as name and patient identification number, has been removed. In The Netherlands, there is no need to obtain consent to make use of registries when patient identifying information is not used. According to the Dutch Medical Research Involving Human Subjects Act, there is no need for approval by ethical committees [12]. The NICE initiative is officially registered according to the Dutch Personal Data Protection Act.

The variables are used to calculate probabilities of death for each patient using the Acute Physiology and Chronic Health Evaluation (APACHE) II [13], the Simplified Acute Physiology Score (SAPS) II [14], and the Mortality Probability Models (MPM) II at admission and 24-h scoring systems [15]. In this study the SAPS II was used for case mix adjustment as previous research has shown that this scoring system fits best to the patient population of the NICE registry [16]. The database contains 108 demographic, diagnostic and physiologic variables collected within the first 24 h of ICU admission and outcome data on ICU and in-hospital mortality.

In analogy with the exclusion criteria commonly used in analyses based on the SAPS II scoring system, patients admitted after cardiac surgery, patients admitted with severe burns and patients aged under 18 were excluded from the analyses. For patients with multiple ICU admissions during a hospitalisation period only the first ICU admission was used.

In the analyses focusing on oxygenation in the first 24 h of ICU stay, information of all patients was used. For the analyses related to oxygenation during the entire ICU stay a selection of the patients was used, as only five of the ICUs participating in the NICE registry provide information to the registry database on the patient's condition on a daily basis using the Sequential Organ Failure Assessment (SOFA) score [17]. For this analysis only patients with a minimum length of ICU stay of 3 days were included into the analyses. Mean PaO_2 _and mean FiO_2 _values were calculated based on the entire ICU stay.

If more than one blood gas analysis was available for a patient during the first 24 h after ICU admission, PaO_2_, FiO_2 _and partial CO_2 _(PaCO_2_) values were from the arterial sample with the lowest PaO_2_/FiO_2 _ratio. Likewise, oxygenation data in the SOFA scores was based on samples with lowest PaO_2_/FiO_2 _ratios in the particular 24 h period.

### Statistical analyses

The relation between the oxygenation parameters and in-hospital mortality was assessed using logistic regression analysis.

As PaO_2 _and FiO_2 _are both continuous variables, univariate regression analyses using polynomial functions and spline functions [18,19] were performed to investigate the relation between each of these variables and in-hospital mortality. For PaO_2 _a model including PaO_2 _as a natural spline with five degrees of freedom turned out to result in the best fit. To enhance interpretation of the results, in subsequent analyses PaO_2 _was categorised. As no standard cut-off points are in use for PaO_2_, categorisation of PaO_2 _values into five categories was based on the distribution of the data, using quintiles as cut-off values between categories. For FiO_2 _inclusion into the model in a linear fashion showed to be optimal fit.

Multivariable logistic regression analyses were performed both for the first 24 h of ICU stay and for the entire ICU stay. Potential confounders in the association of oxygenation with hospital mortality (age, SAPS II, Glasgow Coma Scale (GCS) score below 15 and admission type) were included into the models. Also a specific variable denoting the 'hospital' was included into these models to correct for potential differences in overall in-hospital mortality between the five hospitals. In the model focusing on the entire ICU stay, the PaO_2_/FiO_2 _ratio during the first 24 h of admission was added as additional confounder. In the modelling process the presence of multicolinearity between the oxygenation parameters was verified based on the standard errors of the parameters in the model.

The Standardised Mortality Ratio (SMR) was calculated as the ratio of the number of observed deaths to the number of deaths expected according to the SAPS II model [13].

In all analyses a p value of 0.05 was considered to represent a statistically significant difference. The analyses were performed using SPSS version 14.0 (SPSS Inc., Chicago, IL, USA) and S-plus version 7.0 (Insightful Corp., Seattle, WA, USA).

## Results

### Analysis of data from first 24 h after admission

**Figure 1:**
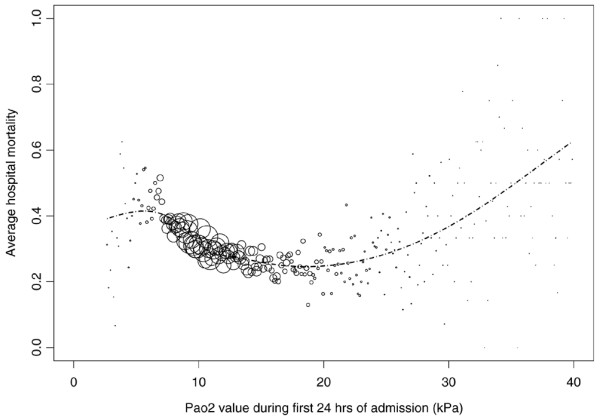
**In-hospital mortality by partial oxygen pressure (PaO_2_) (kPa)**. Values were taken from blood gas analysis with lowest PaO_2_/fraction of oxygen in inspired air (FiO_2_) ratio in the first 24 h after intensive care unit (ICU) admission. The sizes of the circles represent the number of patients with the same PaO_2 _value. The curve represents the predicted mortality using the logistic regression equation in which the PaO_2 _value was incorporated using a spline function.

**Figure 2:**
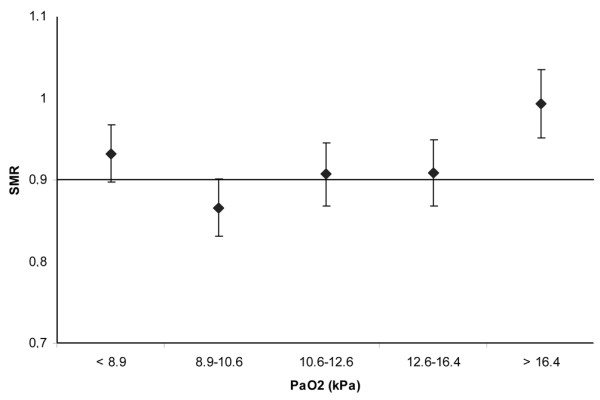
**Standardised mortality ratio (SMR) by partial oxygen pressure (PaO_2_) (kPa)**. PaO_2 _values were taken from blood gas analysis with lowest PaO_2_/fraction of oxygen in inspired air (FiO_2_) ratio in the first 24 h after intensive care unit (ICU) admission. PaO_2_ values are categorised as quintiles. Error bars represent 95% confidence intervals.

**Figure 3:**
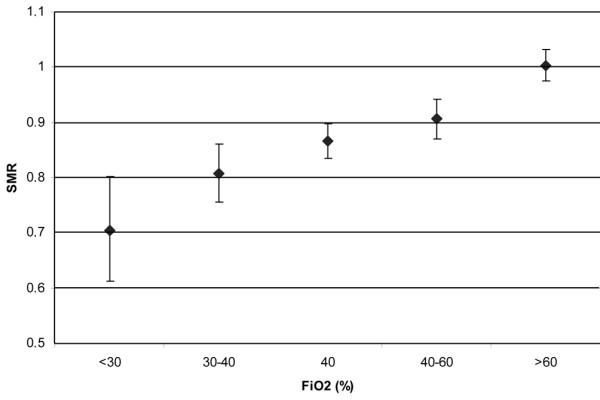
**Standardised mortality ratio (SMR) by fraction of oxygen in inspired air (FiO_2_)**. FiO_2 _values were taken from blood gas analysis with lowest partial oxygen pressure (PaO_2_)/FiO_2 _ratio in the first 24 h after intensive care unit (ICU) admission FiO_2 _values are categorised as quintiles. Error bars represent 95% confidence intervals.

**Figure 4:**
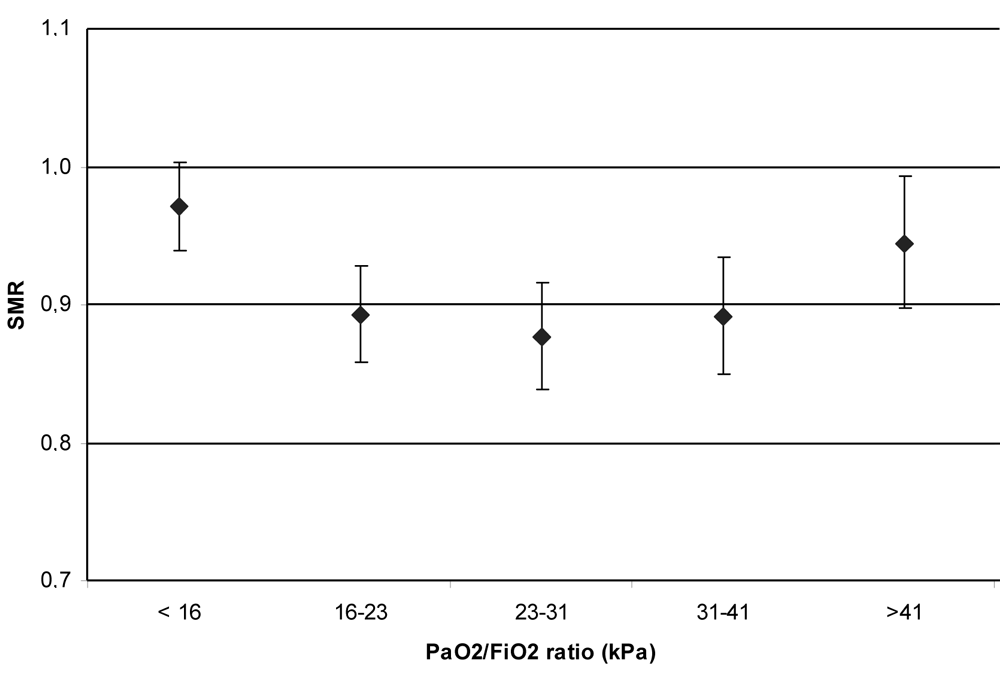
**Standardised mortality ratio (SMR) by lowest partial oxygen pressure (PaO_2_)/fraction of oxygen in inspired air (FiO_2_) ratio (kPa) in the first 24 h after intensive care unit (ICU) admission**. PaO_2_/FiO_2 _ratio values are categorised as quintiles. Error bars represent 95% confidence intervals.

In total, 36,307 patients from 50 ICUs were included in the analysis. All patients were treated with mechanical ventilation within the first 24 h after ICU admission. Data on the severity of illness, reason for admission and referring specialty are given in Table 1. Mean PaO_2 _was 13.2 kPa (standard deviation (SD) 6.5). Mean FiO_2 _was 50% (SD 20). Regression analysis using PaO_2 _as a continuous variable showed that with increasing PaO_2 _the average in-hospital mortality first decreased and subsequently started to rise again (Figure 1). Figure 2 denotes the relation between PaO_2 _and in-hospital mortality when correcting for SAPS II (by means of the SMRs). Figures 3 and 4 show the association between SMR and FiO_2 _or PaO_2_/FiO_2 _ratio respectively. Multivariate regression analysis indicated that the U-shaped relation between PaO_2 _and mortality (modelled using a spline function) remained significant after correction for age, admission type, GCS score and severity of illness measured with the SAPS II. Multicolinearity was not found to be present for FiO_2 _and PaO_2 _values. Table 2 presents the odds ratios for FiO_2 _and the PaO_2 _quintiles in a multivariate regression model, indicating that in-hospital mortality was associated with FiO_2 _and PaO_2 _values.

**Table 1:** Characteristics of patients

	**Analysis of data from first 24 h after admission**	**Analysis of data from all admission days**
No of patients	36,307	3,322
Male (%)	60.1	60.3
Age in years^a^	62.5 ± 16.1	62.4 ± 15.9
SAPS II^a^	42.7 ± 18.4	47.6 ± 15.7
SAPS II predicted mortality^a^	0.34 ± 0.28	0.42 ± 0.27
GCS score below 15 (%)	33.1	33.6
PaO_2 _at admission (kPa)^a^	13.2 ± 6.5^b^	12.5 ± 5.5^c^
FiO_2 _(%)^a^	50.4 ± 19.9^b^	53.1 ± 18.7^c^
PaO_2_/FiO_2 _ratio (kPa)^a^	29.1 ± 15.0^b^	24.6 ± 12.6^c^
Admission type (%):		
Medical	48.4	61.0
Unplanned surgery	22.6	23.0
Planned surgery	28.9	16.0
Referring specialty (%):		
Internal medicine	16.4	16.7
Cardiology	10.8	17.9
Pulmonary disease	7.4	10.4
Neurology	5.7	6.4
Surgery	33.2	30.6
Cardiothoracic surgery	6.4	1.2
Neurosurgery	6.9	6.1
Other	13.1	10.7
ICU mortality	23.0	21.0
In-hospital mortality (%)	31.1	32.7

^a^Mean ± standard deviation (SD); ^b^PaO_2_, FiO_2 _and PaO_2_/FiO_2 _ratio from sample with lowest PaO_2_/FiO_2 _ratio within 24 h after admission; ^c^mean value of all admission days. Per day PaO_2_, FiO_2 _and PaO_2_/FiO_2 _ratio was taken from sample with lowest PaO_2_/FiO_2 _ratio.

GCS, Glasgow Coma Scale; FiO_2_, fraction of oxygen in inspired air; ICU, intensive care unit; PaO_2_, partial oxygen pressure; SAPS II, Simplified Acute Physiology Score II.

**Table 2:** Adjusted odds ratios for partial oxygen pressure (PaO_2_) and fraction of oxygen in inspired air (FiO_2_) resulting from a multivariate regression analysis on data from the first 24 h after ICU admission

**Covariate**	**Odds ratio**	**95% Confidence interval**
PaO_2 _in kPa:		
< 8.9 (n = 6,937)	1.12	1.03 to 1.21
8.9 to 10.6 (reference category) (n = 7,466)	1	
10.6 to 12.6 (n = 6,430)	1.11	1.02 to 1.21
12.6 to 16.4 (n = 7,278)	1.08	1.00 to 1.18
≥ 16.4 (n = 8,196)	1.23	1.13 to 1.34
FiO_2 _(per 10%)	1.12	1.10 to 1.13

Odds ratio after adjustment for the following potential confounders: age, SAPS II, GCS score below 15, admission type, individual hospital. The equation of the model is: Logit(p) = -5.419 + 0.059 × age (per 5 years) + 0.066 × SAPS II + 0.070 × *I*(GCS < 15) + 0.221 × *I*(admission type = urgent) + 0.453 × *I*(admission type = medical) + β_hosp _+ 0.105 × FiO_2 _(per 10%) + 0.109 × *I*(PaO_2 _< 67) + 0.109 × *I*(80 ≤ PaO_2 _< 95) +0.079 × *I*(95 ≤ PaO_2 _< 123) +0.206 × *I*(PaO_2 _≥ 123) Probability of in-hospital death = e^(logit)^/(1+e^(logit)^). Median β_hos _for individual hospitals was -0.12 (IQR -0.43 to 0.05).

GCS, Glasgow Coma Scale; ICU, intensive care unit; IQR, interquartile range; SAPS II, Simplified Acute Physiology Score II.

### Analysis of data from all ICU admission days

For the analysis regarding entire ICU stay 3,322 patients from 5 ICUs were included. Characteristics of these patients are given in Table 1. Mean PaO_2 _during ICU stay was 12.4 kPa (SD 5.5). Mean FiO_2 _during ICU stay was 53% (SD 18). The results of the multivariate analysis are shown in Table 3. Mean FiO_2 _value and a mean PaO_2 _value lower than 10.6 kPa were associated with a higher mortality. This association was independent of the potential confounders age, SAPS II, abnormal GCS score, PaO_2_/FiO_2 _ratio at admission, admission type and hospital.

**Table 3:** Adjusted odds ratios for mean partial oxygen pressure (PaO_2_) value and mean fraction of oxygen in inspired air (FiO_2_) during ICU stay resulting from a multivariate regression analysis on data from the entire ICU stay

**Covariate**	**Odds ratio**	**95% Confidence interval**
Mean PaO_2 _in kPa:		
< 8.9 (n = 402)	1.63	1.16 to 2.3
8.9 to 10.6 (n = 871)	1.51	1.18 to 1.96
10.6 to 12.6 (n = 970)	1.25	0.99 to 1.57
12.6 to 16.4 (reference category) (n = 841)	1	
> 16.4 (n = 238)	1.04	0.64 to 1.68
Mean FiO_2 _(per 10%)	1.63	1.47 to 1.81

Odds ratio after adjustment for the following potential confounders: age, SAPS II, GCS score below 15, admission type, PaO_2_/FiO_2 _ratio at admission, and hospital. The equation of the eventual model is: Logit(p) = -7.060 + 0.090 × age (per 5 years) + 0.049 × SAPS II + 0.054 × *I*(GCS < 15) + 0.015 × *I*(admission type = urgent) + 0.161 × *I*(admission type = medical) + 0.004 × PaO_2_/FiO_2 _– 1.114 × I(hospital = 2) – 0.060 × I(hospital = 3) – 0.285 × I(hospital = 4) – 0.618 × I(hospital = 5) + 0.488 × mean FiO_2 _(per 10%) + 0.492 × *I*(mean PaO_2 _< 67) + 0.417 × *I*(67 ≤ PaO_2 _< 80) + 0.221 × *I*(80 ≤ PaO_2 _< 95) + 0.038 × *I*(PaO_2 _≥ 123). Probability of in-hospital death = e^(logit)^/(1+e^(logit)^).

GCS, Glasgow Coma Scale; ICU, intensive care unit; SAPS II, Simplified Acute Physiology Score II.

## Discussion

We found that administration of high FiO_2 _values in ICU patients was associated with increased in-hospital mortality. This association was found for FiO_2 _values in the first 24 h after admission and also for mean FiO_2 _during all admission days. The increased risk in patients with high FiO_2 _remained after correcting for SAPS II, admission type, reduced GCS score and pulmonary dysfunction measured as PaO_2_/FiO_2 _ratio. This suggests that the administration of oxygen itself could be deleterious, and that the association between high FiO_2 _and mortality cannot be explained by the confounding issue that highest FiO_2 _levels are administered in patients with severe pulmonary dysfunction.

Our observations are in accordance with prior experimental studies showing the potential toxicity of high fractions of inspired oxygen [5]. Administration of supplemental oxygen can cause lung damage. This risk is especially high in prematurely born infants, probably attributable to inadequate host defences, underdeveloped lungs and immature antioxidant systems [20]. Exposure to hyperoxia leads to diffuse pulmonary damage characterised by an extensive inflammatory response and destruction of the alveolar-capillary barrier leading to oedema, impaired gas exchange and respiratory failure [21]. Mouse lungs exposed to > 90% oxygen for 48 h were more susceptible to ventilator-induced lung injury than those exposed to room air [22]. Hyperoxia also aggravates pulmonary injury following artificial ventilation in rats using high tidal volumes [23]. Furthermore, hyperoxia impairs the innate immune response by decreased macrophage function, impaired bacterial killing and increased susceptibility to pneumonia in a *Klebsiella pneumoniae *model [24]. Lung injury is likely to be initiated when the rates of generation of reactive oxygen species (ROS) are increased beyond the capacities of the antioxidant defences, such as the enzymes glutathione, superoxide dismutase and catalase. Mitochondrial mediated cell injury by ROS has been identified as a critical event in both apoptotic and necrotic forms of cell death in hyperoxia [25]. Another organ that may be injured by hyperoxia is the kidney. Hyperoxic reperfusion exacerbates renal dysfunction and histopathologic injury after 30 min of complete normothermic ischaemia in rabbits. This hyperoxia associated dysfunction was prevented by the administration of the radical scavenger allopurinol [26], suggesting that oxidative injury by ROS plays a role in post-ischaemic renal failure.

Several studies focused on the role of high reperfusion oxygen tensions following cardiac arrest and resuscitation. In a canine model of 10 min of cardiac arrest, resuscitation with 21% vs 100% inspired O_2 _resulted in lower levels of oxidised brain lipids and improved neurological outcome [27]. In another study using the same canine model, it was shown that resuscitation with 100% O_2 _resulted in impaired hippocampal neuronal metabolism [28]. Proposed pathogenetic mechanisms of hyperoxia induced reperfusion injury of the brain include increased production of ROS, a high ratio of oxidised over reduced glutathione [29] and increased nitric oxide production by endothelium and neuron derived nitric oxide synthase [30].

Many studies investigated the use of 100% vs 21% oxygen for resuscitation in depressed newborn infants (that is, infants with apnoea or relative bradycardia at birth). A systematic review and meta-analysis of 10 studies reported a significant reduction in the risk of neonatal mortality and a trend towards a reduction in severe encephalopathy in newborns resuscitated with 21% O_2_. The reduction in mortality was also found in a subgroup analysis only including strictly randomised controlled trials and in a subgroup of studies enrolled in European countries with a lower risk of mortality than in less developed countries [31].

Human clinical studies evaluating the effects of hyperoxia in critically ill adult patients are lacking. The effects of hyperoxia in non-ICU settings are not clear. A reduction in surgical site infections by the use of hyperoxia has been reported by one study group [32], while others reported more surgical site infections in patients treated with hyperoxia [33].

An alternative explanation for the association between oxygenation and mortality in ICU patients could be that common criteria for weaning from mechanical ventilation are based on FiO_2 _and PEEP levels. High FiO_2 _and PEEP, both leading to high PaO_2 _values, may delay weaning from mechanical ventilation, thus negatively influencing outcome in ICU patients. Also, we cannot exclude that high PaO_2 _values were achieved by more invasive ventilation strategies, potentially being more injurious to the patients.

Interestingly, apart from FiO_2 _values, there was also a U-shaped association between achieved arterial oxygen tension (PaO_2_) during the first 24 h after ICU admission and mortality with higher mortality in patients with either a very low or high PaO_2_. That mortality is higher in patients with very low PaO_2 _is not unexpected and possibly related to ischaemia or to selection of the sickest patients. However, mortality was also higher in patients with highest PaO_2 _values, suggesting the possibility of systemic oxygen toxicity.

In our analysis of mean oxygenation during all admission days, we again found a linear association between mortality and FiO_2 _values. Low PaO_2_s were also associated with higher mortality but high PaO_2_s were not. The shape of the association between PaO_2 _and mortality was hard to assess. In our data a linear association appeared to best fit the data (data not shown). The number of patients included in this analysis was only 3,322. Only 2% of the patients had a mean PaO_2 _higher than 20.0 kPa. Thus, the power of our study may have been too low to detect an association between high mean PaO_2 _values during the ICU stay and increased mortality.

There are limitations to this study. Most importantly, it was a retrospective observational study and the association between mortality and oxygenation is not necessarily causal. Although the association appeared to be independent of a number of potential confounding covariates, we cannot exclude that, despite our efforts, there are still differences in case mix associated with oxygenation that are not taken into account in our multivariate analyses. It is possible that physicians recognised some marker of severity that was not represented in our attempts to adjust for severity, and that they purposefully gave higher concentrations of oxygen to achieve higher levels of PaO_2 _in these high-risk patients.

The three potential confounders that we corrected for (age, reduced GCS score, and admission type) are part of the SAPS II that was also included as covariate in the multivariate analysis. We have repeated the analyses without these three variables, adjusting for SAPS II only. This yielded similar results for the association between in-hospital mortality and PaO_2 _and FiO_2 _respectively.

We corrected for pulmonary dysfunction by including PaO_2_/FiO_2 _ratio at admission in the multivariate analysis of data from all admission days. PaO_2_/FiO_2 _ratio was not included in the analysis of data from the first 24 h after ICU admission, because including PaO_2_, FiO_2 _and PaO_2_/FiO_2 _ratio, all from the same arterial blood sample, would introduce problems by colinearity of the data. In this population, however, we performed a separate multivariate analysis substituting PaO_2_/FiO_2 _ratio for PaO_2 _values. Again, FiO_2 _appeared to be a predictor of mortality, also independent of PaO_2_/FiO_2 _ratio (OR 1.15, 95% CI 1.14 to 1.17, model not shown). PaO_2_/FiO_2 _ratio is not only influenced by pulmonary dysfunction, but also by ventilator settings, such as PEEP levels. As PEEP was not part of the NICE data collection, we could not include this possible confounder in our analysis. Prospective, controlled trials are necessary to show a causal relationship between high FiO_2_s and mortality.

As the association between PaO_2 _and mortality was U-shaped, we categorised PaO_2 _values for the multivariate analysis using quintiles as categories (as no standard categorisation is available). The boundaries of quintiles are chosen arbitrarily and may not be the optimal cut-off levels to discriminate between patients with low and high risk of mortality. Therefore, we repeated the same multivariate analysis (model not shown) on data from the first 24 h after ICU admission using PaO_2 _values categorised as deciles and found similar results.

Another finding from our study is the fact that in most patients the achieved PaO_2 _values are higher than the targets commonly recommended [2,4]. Although oxygen toxicity is a well known entity [34], FiO_2_s up to 0.5 are commonly considered 'safe' by physicians [5]. It appears that physicians are more concerned about avoiding hypoxia and ischaemia than about the risks of hyperoxia. In The Netherlands, no formal guidelines for oxygenation targets are available. This may be related to the fact that the influence of oxygenation targets has never been studied making it impossible to provide evidence-based recommendations. Based on other observational studies, it may well be that also in other countries actual PaO_2_s in ICU patients are higher than recommended [35,36]

## Conclusion

High fractions of oxygen in the inspired air and high PaO_2 _values are associated with increased mortality in ICU patients. Actually achieved PaO_2 _values in Dutch ICU patients are higher than the PaO_2 _targets in some recent international recommendations. Prospective interventional studies are necessary to find out whether the association between outcome and oxygenation is causal and to provide evidence-based guidelines on oxygenation targets.

## Key messages

• The weaning rate of catecholamines is usually chosen empirically by intensivists.

• Actually achieved PaO_2 _values in Dutch ICU patients are higher than the PaO_2 _targets given in recent international recommendations.

• High fractions of oxygen in the inspired air are associated with increased mortality in ICU patients on mechanical ventilation.

• Both low and high PaO_2 _values in the first 24 hours after ICU admission were associated with increased mortality.

• Future interventional studies are required to find out whether these associations between oxygenation and outcome are causal or due to other confounding issues.

## Abbreviations

APACHE II: Acute Physiology and Chronic Health Evaluation II; FiO_2_: fraction of oxygen in the inspired air; MPM II: Mortality Prediction Model II; NICE: National Intensive Care Evaluation; PaO_2_: partial pressure of oxygen; SAPS II: Simplified Acute Physiology Score II; SOFA: Sequential Organ Failure Assessment.

## Competing interests

During the period from 2002 to 2004 LP received an unrestricted educational grant from Eli Lilly Netherlands B.V. The study described in this manuscript was not conducted under the grant, and Eli Lilly Netherlands B.V. has not been involved in any part of the present study. All other authors declare that they have no competing interests.

## Authors' contributions

EdJ designed the study and drafted the manuscript. LP and NdK were involved in the set-up of the study, performed the statistical analyses and helped in interpreting the results and writing the manuscript. PK was involved in the set-up of the study, interpreting the results and writing the manuscript. JJ, DdL, PvdV, RB, RdW and RW were involved in interpreting the results and writing the manuscript. All authors read and approved the final manuscript.
